# Porphyrin-lipid nanovesicles (Porphysomes) are effective photosensitizers for photodynamic therapy

**DOI:** 10.1515/nanoph-2021-0220

**Published:** 2021-06-22

**Authors:** Keegan Guidolin, Lili Ding, Juan Chen, Brian C. Wilson, Gang Zheng

**Affiliations:** Department of Surgery, University of Toronto, Toronto, ON, Canada; Princess Margaret Cancer Centre, Toronto, ON, Canada; Institute of Biomedical Engineering, University of Toronto, Toronto, ON, Canada; Department of Medical Biophysics, University of Toronto, Toronto, ON, Canada

**Keywords:** cancer, nanomedicine, nanoparticles, photodynamic therapy, photofrin, porphysome

## Abstract

Porphysomes (PS) are liposome-like nanoparticles comprising pyropheophorbide-conjugated phospholipids that have demonstrated potential as multimodal theranostic agents for applications that include phototherapies, targeted drug delivery and *in vivo* fluorescence, photoacoustic, magnetic resonance or positron emission imaging. Previous therapeutic applications focused primarily on photothermal therapy (PTT) and suggested that PSs require target-triggered activation for use as photodynamic therapy (PDT) sensitizers. Here, athymic nude mice bearing subcutaneous A549 human lung tumors were randomized into treatment and control groups: PS-PDT at various doses, PS-only and no treatment negative controls, as well as positive controls using the clinical photosensitizer Photofrin. Animals were followed for 30 days post-treatment. PS-PDT at all doses demonstrated a significant tumor ablative effect, with the greatest effect seen with 10 mg/kg PS at a drug-light interval of 24 h. By comparison, negative controls (PS-only, Photofrin-only, and no treatment) showed uncontrolled tumor growth. PDT with Photofrin at 5 mg/kg and PS at 10 mg/kg demonstrated similar tumor growth suppression and complete tumor response rates (15 vs. 25%, *p* = 0.52). Hence, porphysome nanoparticles are an effective PDT agent and have the additional advantages of multimodal diagnostic and therapeutic applications arising from their intrinsic structure. Porphysomes may also be the first single all-organic agent capable of concurrent PDT and PTT.

## 1 Introduction

Porphysomes (PSs) are nanoparticles (∼100 nm) composed of pyropheophorbide-conjugated phospholipid (pyro-lipid) subunits that self-assemble into liposome-like structures, each containing ∼80,000 porphyrin molecules [1]. They are preferentially taken up and retained in solid tumors via the enhanced permeability and retention (EPR) effect and subsequently taken up by cancer cells and disrupted into pyro-lipid subunits [1]. Previous studies have suggested that the PSs can be exploited in both their structurally intact state (i.e., as nanoparticles) and in their disrupted state (i.e., as pyro-lipid subunits). In the former state, absorption of light energy is dissipated through thermal conversion and no significant fluorescence emission or photochemistry occurs due to the tight packing of porphyrin molecules in the porphyrin-lipid bilayer, resulting in a quenching of >99% [1], [2], [3], [4], [5], [6], [7]. Upon disruption, the pyro-lipid subunits are unquenched, enabling fluorescence and photochemical reactions [2, 4, 7, 8]. These previous studies suggest that the conversion of PSs from their intact to their disrupted state is time dependent, though the relative populations of intact and disrupted PSs at any given time point are not well characterized.

PSs in various formulations have been shown to be effective multimodality agents for theranostic applications, including *in vivo* photoacoustic imaging and photothermal therapy (PTT) in the intact state, fluorescence imaging after unquenching, and positron emission tomography via ^64^Cu chelation or magnetic resonance imaging via Mn chelation in either state [1], [2], [3], [4], [5], [6], [7], [8]. Disrupted PSs have also been shown to produce reactive oxygen species upon irradiation at the main 671 nm pyro absorption wavelength, providing a rationale for their use as photodynamic sensitizers [8]. PSs conjugated to folate have been used to deliver target-triggered PDT in different tumor models. Limited prior studies of the untargeted PSs showed low PDT efficacy; however, these studies were neither systematic nor optimized [2], [8].

**Figure 1:**
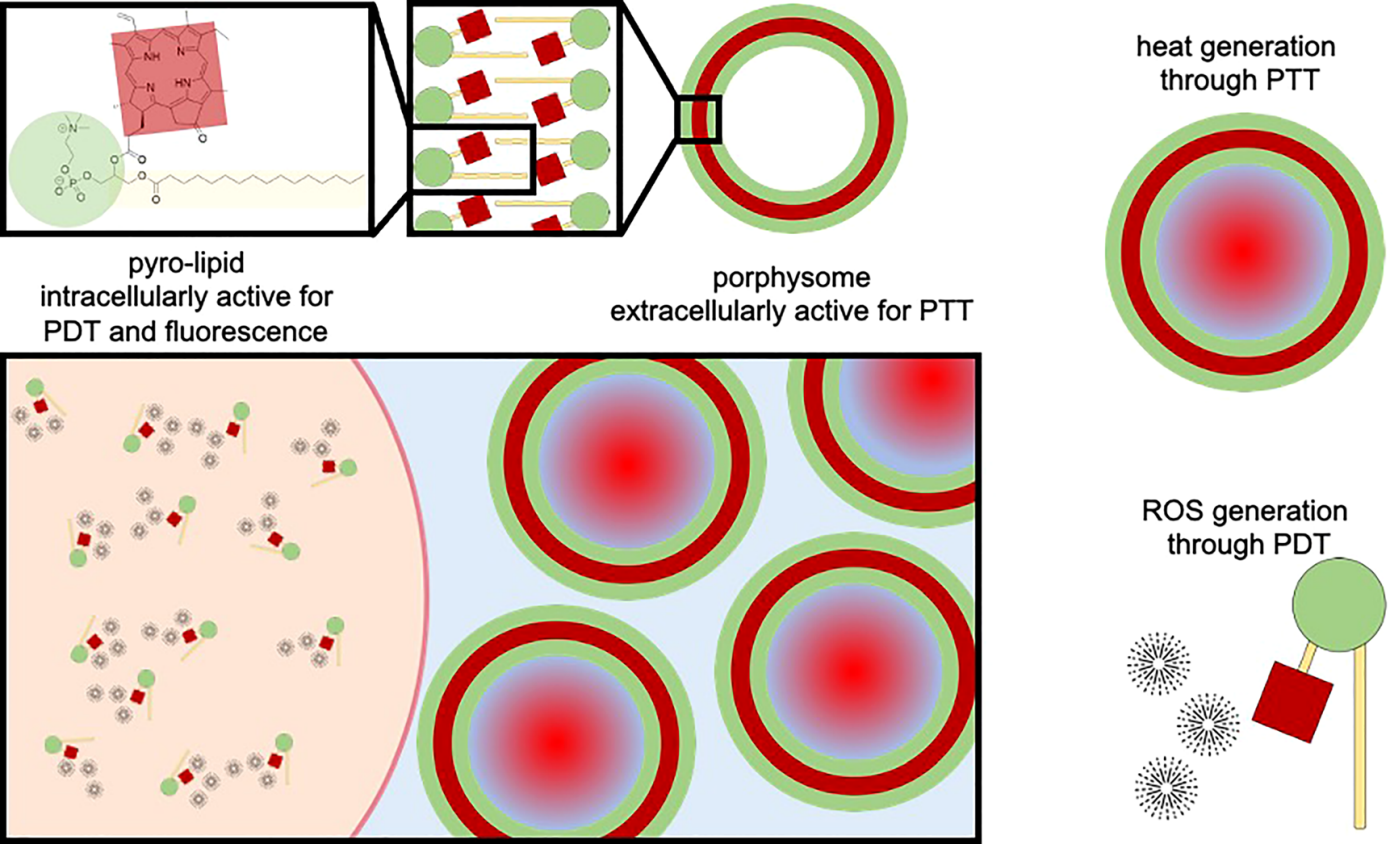
Schematic diagram of the distribution and activity of porphysome nanoparticles and dissociated pyro-lipid in the tumor. When irradiated with 671 nm light, the intact porphysomes in the extracellular space respond photothermally (lower panel, right), while the disrupted intracellular porphysomes (pyro-lipid) are unquenched and generate reactive oxygen species (ROS; e.g., ^1^O_2_) for photodynamic therapy (lower panel, left).

Since the activation wavelength for both PS-PTT and PS-PDT is 671 nm, PS holds the potential to be the first single agent, single wavelength, all-organic photosensitizer that can accomplish both PTT and PDT using a dual population of intact PSs in the extracellular matrix and disrupted PSs in the intracellular space (Figure 1). It must first be established that untargeted PSs can accomplish PDT. Hence, the objectives of the present study were to test if PSs can be used as a practical PDT agent, to determine the optimum treatment conditions for this, and to compare PS-PDT to PDT using Photofrin (PHO, Pinnacle Biologics, Bannockburn, IL, USA), a long-established, clinically-approved, porphyrin-based PDT agent [9], [10], [11], [12].

## 2 Materials and methods

### 2.1 Cell culture and animal models

A549 human lung cancer cells (American Type Culture Collection, Manassas, VA, USA) were cultured in F12K media supplemented with fetal bovine serum at a final concentration of 10% and prepared for injection in a 1 : 1 mixture of phosphate buffered saline and Matrigel (Corning Inc., Corning, NY, USA). Female Hsd:athymic nude *Foxn1* ^*nu*^ mice (Envigo, Indianapolis, IN, USA), aged 8–10 weeks, underwent subcutaneous injection of 5 × 10^6^ A549 cells in the right shoulder. Tumor growth was monitored and, upon reaching 5 mm in the maximum dimension, the mice were randomized into various treatment and control groups. An *a priori* humane endpoint of 500 mm^3^ maximum tumor volume was set. All animal studies were conducted in compliance with institutional guidelines (University Health Network, Toronto, Canada).

### 2.2 Photosensitizer and light administration

Porphysomes were prepared according to previously published protocols [1]. Photofrin (PHO) was generously provided by Pinnacle Biologics (Bannockburn, IL). Following randomization, photosensitizer was administered intravenously by bolus tail vein injection at an appropriate drug-light interval (DLI) before light irradiation. PS was administered in doses of 5, 7.5 or 10 mg (of pyropheophorbide-lipid) per kg of animal body weight, and PHO was administered at 5 mg/kg of animal body weight; each was diluted with phosphate-buffered saline to a total volume of 250 μl per dose. Diode lasers at 671 and 630 nm were used for PS and PHO activation, respectively (Figures S1 and S2). Under general anesthesia (2–3% inhaled isoflurane) and with minimal ambient lighting, the treatment light was delivered via an optical fiber as a 10 mm diameter circular spot centered on the tumor. The energy density was measured prior to each treatment using a calibrated power meter (Thorlabs Inc., Newton, NJ, USA). A total light dose of 135 J/cm^2^ was delivered at 100 mW/cm^2^ over 1350 s (22.5 min). Animals were protected from scattered light using an opaque mask with a 10 mm diameter hole.

### 2.3 Study metrics

Following treatment, the mice were followed for 30 days and the tumor size was measured using calipers 3 times per week. Photographs of tumors were taken, and the animals were weighed on each occasion. Humane sacrifice by anesthetic overdose was carried out after 30 days or if tumors reached 500 mm^3^ volume, whichever came first. Animals were considered to have reached a “mortality” endpoint if they required sacrifice because their tumor volume was >500 mm^3^.

### 2.4 Study aims

The first aim of this study was to confirm that irradiation at 100 mW/cm^2^ and the highest planned PS dose (10 mg/kg) and 24 h drug-light interval (DLI) did not produce significant thermal conversion. Hence, in 3 mice the surface temperature of the tumor was measured with a thermal camera (Mikron Infrared, Santa Clara, CA, USA), with an *a priori* maximum endpoint of a 5 °C increase during irradiation. In separate groups, the optimal PS dose and DLI were assessed: 5, 7.5 and 10 mg/kg at 24 h; 10 mg/kg at 48 h; 10 mg/kg, no light; 5 mg/kg PHO, no light; and no drug, no light; (*n* = 5 per group except *n* = 6 for 10 mg/kg, 24 h). For head-to-head comparison of the optimized PS-PDT regimen and PHO-PDT, separate groups were treated with either 10 mg/kg PS at 24 h DLI (*n* = 20) or 5 mg/kg PHO at 24 h DLI (*n* = 20).

### 2.5 Analysis

In each animal, the tumor volume post treatment was normalized to the pre-treatment volume. Complete response was defined as the absence of detectable tumor upon dissection at the end of the 30-day follow-up period (i.e., cure). ANOVA was performed to assess for differences in the relative tumor size between groups. Fisher’s exact test was used to compare cure rates between groups. Log-rank (Mantel-Cox) tests were conducted for comparison of survival curves. Statistical analysis was performed using GraphPad Prism software; *p* < 0.05 were considered significant.

## 3 Results

**Figure 2:**
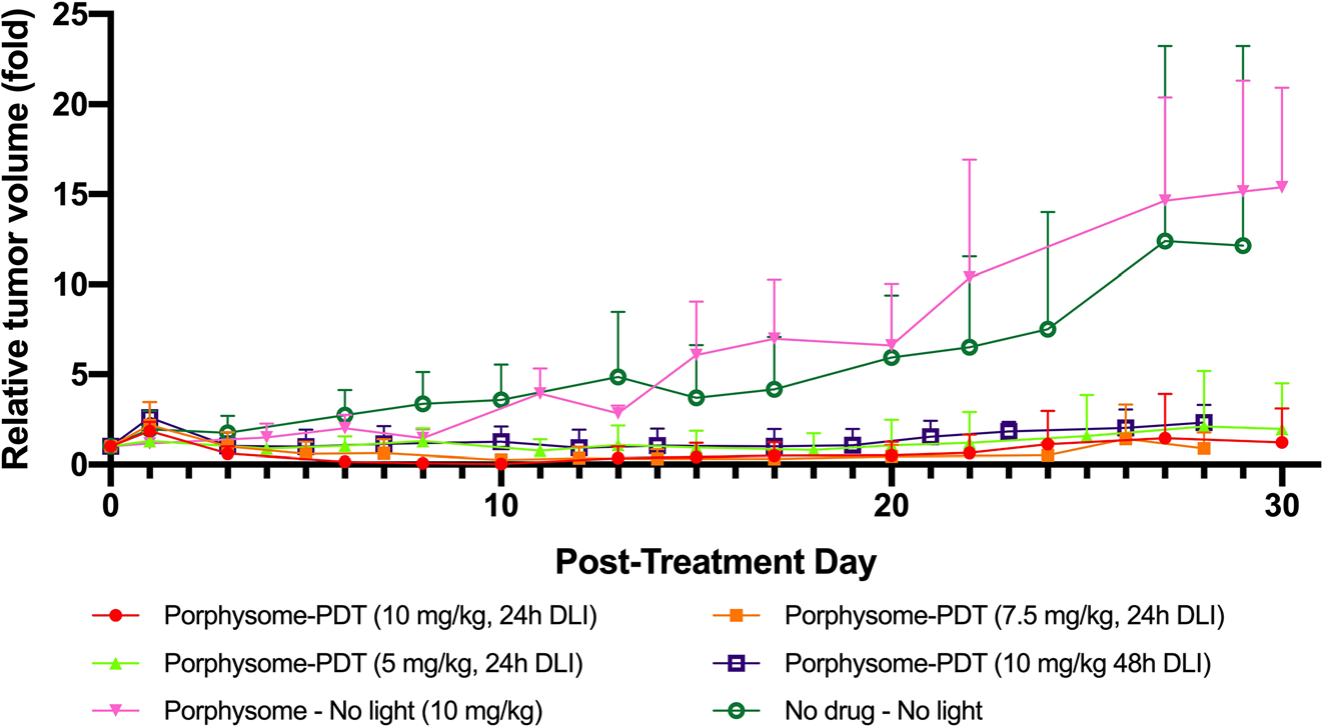
Relative tumor volume across the treatment and control groups in the 30 d post-PDT follow-up period. (*n* = 5 for all groups; except *n* = 6 for the PS 10 mg/kg, 24 h DLI group). Mean ± 1 S.D. with half bars shown for clarity.

**Figure 3:**
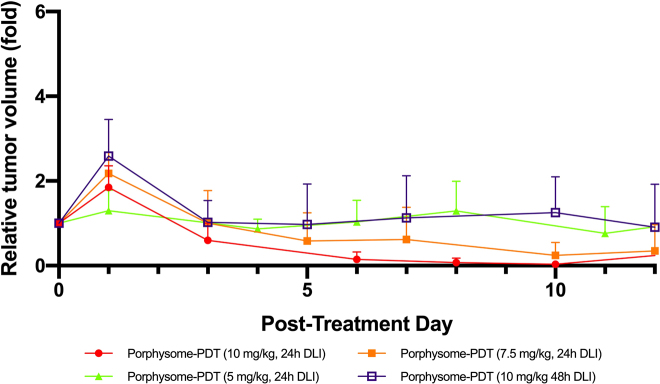
Relative tumor volume across dose-finding groups with 12 days of follow-up. Mean ± 1 S.D. with half bars shown for clarity.

In the first experiment, the maximum increase in tumor surface temperature recorded at any time during treatment (*n* = 3) was 1.8 °C. All PS-PDT treated tumors demonstrated at least partial response, with growth curves that were significantly different from the untreated groups: Figure 2. The strongest response was observed in the group treated with 10 mg/kg PS at 24 h DLI; followed by 7.5 mg/kg at 24 h, 5 mg/kg at 24 h and 10 mg/kg at 48 h. Tumor size was the smallest at 10–15 d post treatment, at which time the distinction between the treatment groups was most evident; tumor regrowth was evident thereafter. Figure 3 compares the tumor responses at each PS dose with follow-up to 12 days to illustrate more clearly the differences between groups.

**Figure 4:**
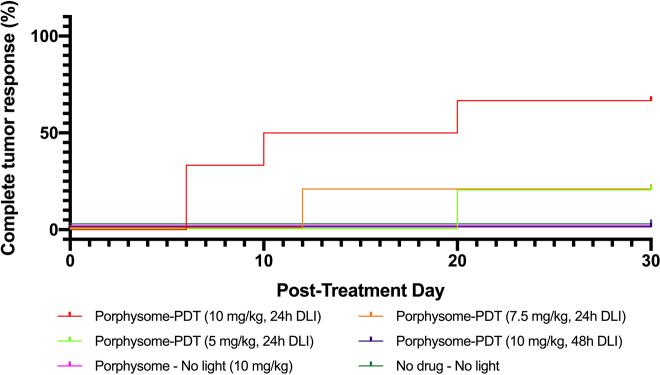
Complete tumor responses for treatment and control groups.

**Figure 5:**
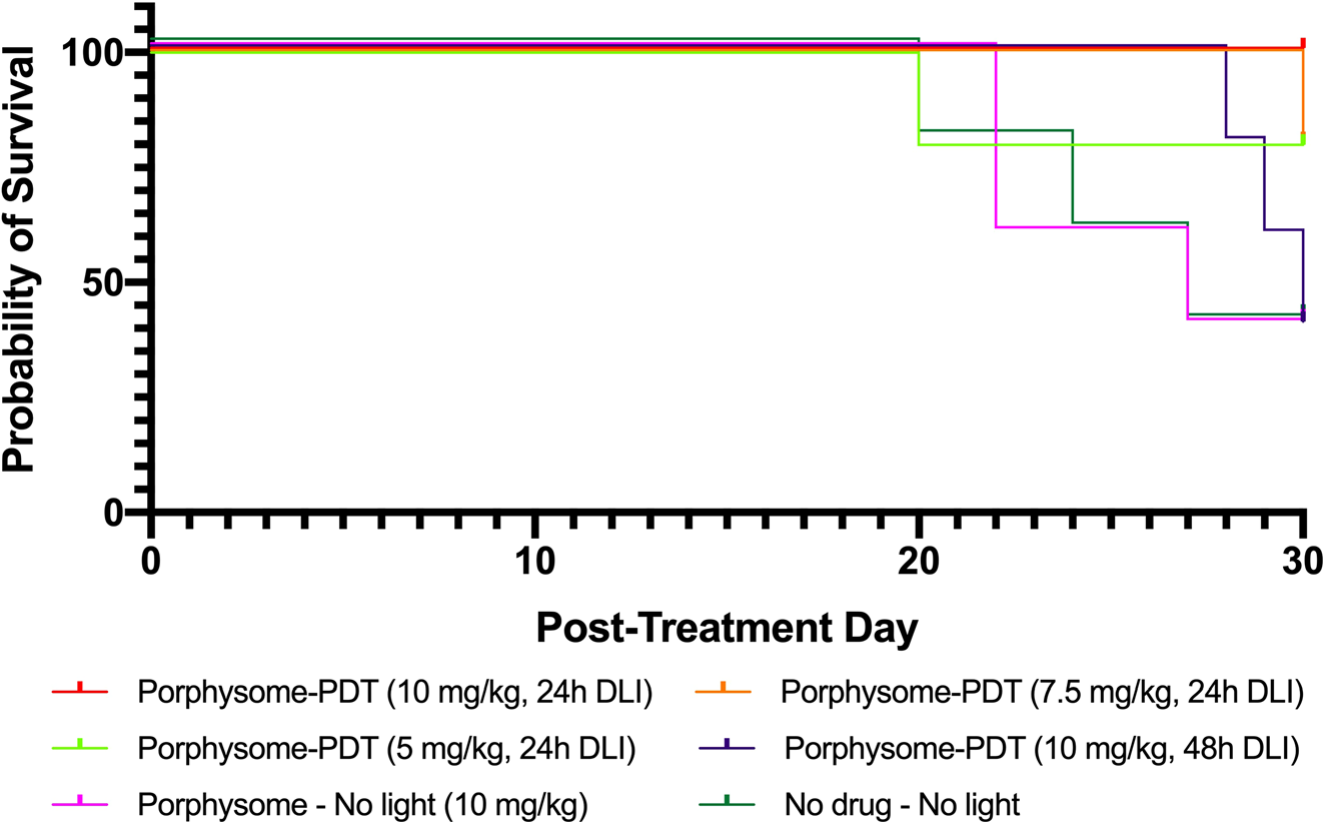
Kaplan–Meier plots of treatment and control groups.

Complete tumor response was achieved in 4/6 cases (67%) with 10 mg/kg PS at 24 h and in 1/5 cases (20%) with 7.5 or 5 mg/kg PS at 24 h. Neither the 10 mg/kg, 48 h DLI treatment group nor any of the control groups achieved complete tumor response in any animals (Figure 4). The only group that achieved 100% survival to 30 days post treatment was for PS-PDT at 10 mg/kg and 24 h DLI. All other groups had at least one mortality in the follow-up period (due to uncontrolled tumor growth, requiring sacrifice): 1/5 cases for 7.5 and mg/kg and 24 h DLI; 3/5 cases for 10 mg/kg and 48 h DLI group; 3/5 cases for the PS, no light; and no treatment groups; and 5/5 cases for the PHO, no light group. The corresponding Kaplan–Meier survival plots are shown in Figure 5.

**Figure 6:**
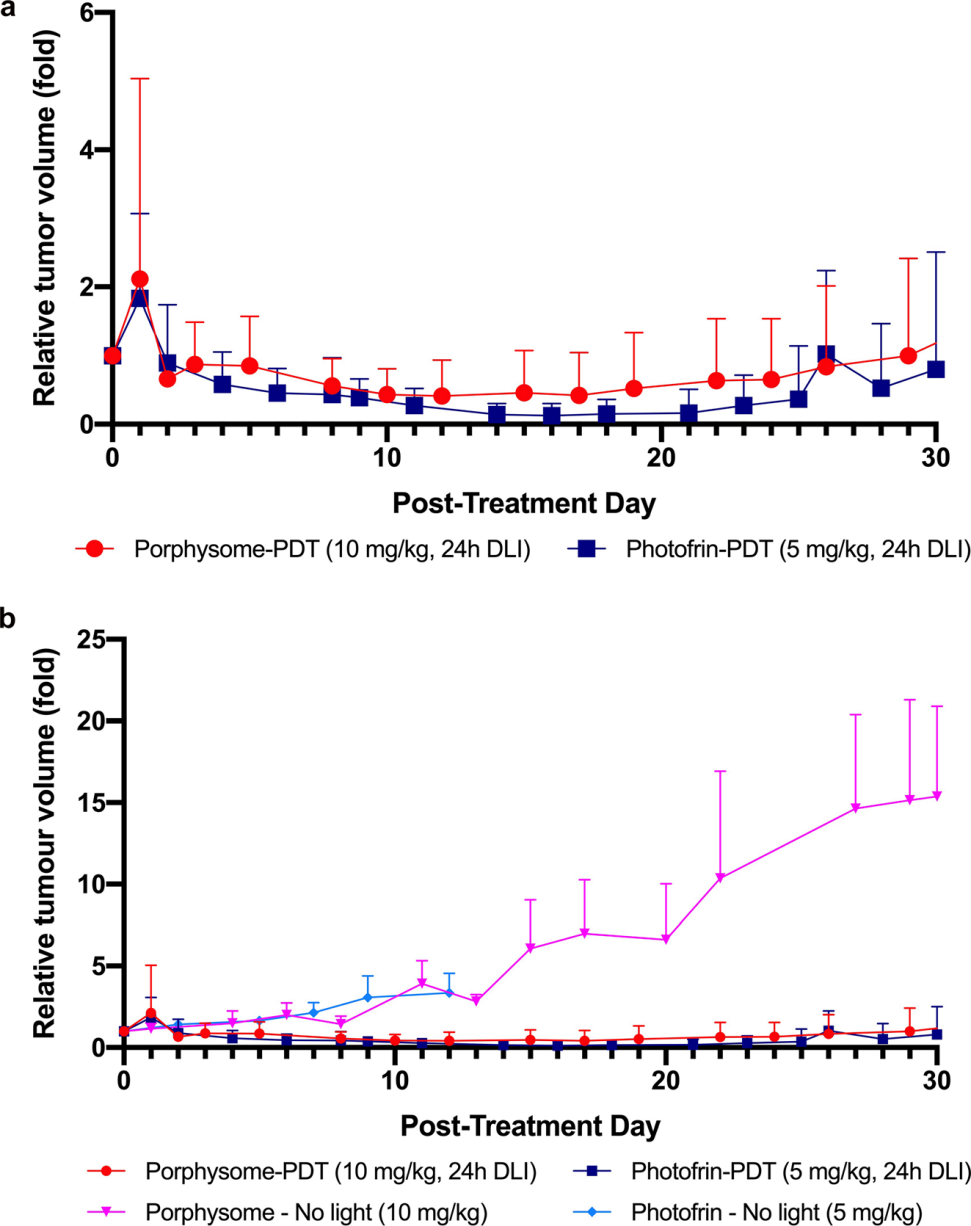
(a) Relative tumor volume for porphysome-PDT (10 mg/kg, 24 h DLI, *n* = 20) and Photofrin-PDT (5 mg/kg, 24 h DLI, *n* = 20) with 30 d follow-up post PDT. Mean ± 1 S.D. with half bars shown for clarity. (b) Relative tumor volume for porphysome-PDT, Photofrin-PDT, porphysome-only (10 mg/kg, no light, *n* = 5), and Photofrin-only (5 mg/kg, no light, *n* = 5) with 30 d follow-up post PDT. Mean ± 1 S.D. with half bars shown for clarity.

**Figure 7:**
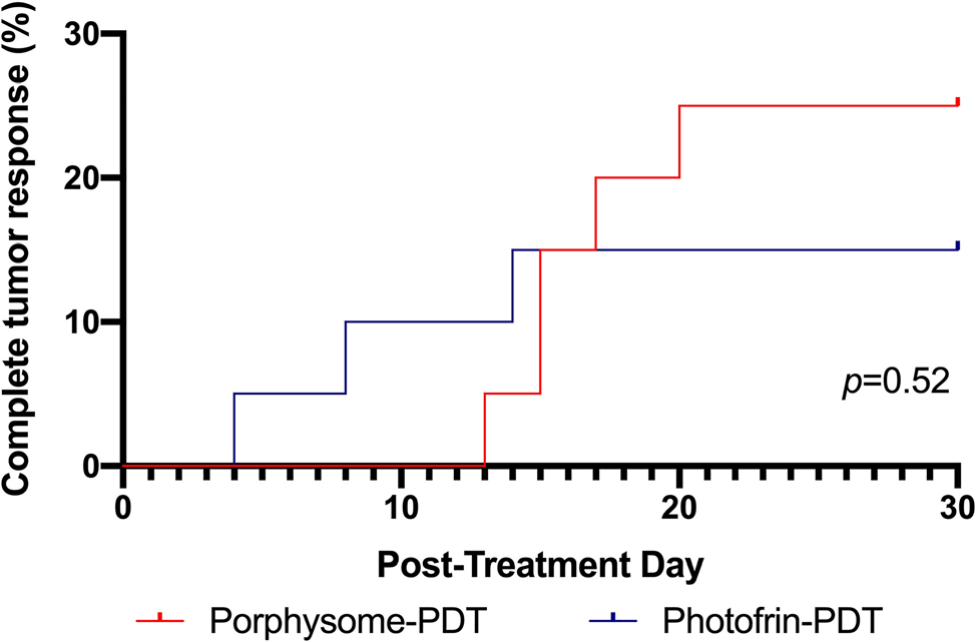
Complete tumor responses in porphysome-PDT (10 mg/kg, 24 h DLI, *n* = 20) and Photofrin-PDT (5 mg/kg, 24 h DLI, *n* = 20).

PS-PDT and PHO-PDT resulted in similar treatment outcomes in terms of delayed tumor growth control (Figure 6), complete tumor response and survival. No animals had to be sacrificed due to tumor size in either group. Complete tumor response was achieved in 5/20 (25%) of PS-PDT treated animals and in 3/20 (15%) of PHO-PDT treated animals (Figure 7). This difference was not statistically significant (*p* = 0.52).

**Figure 8:**
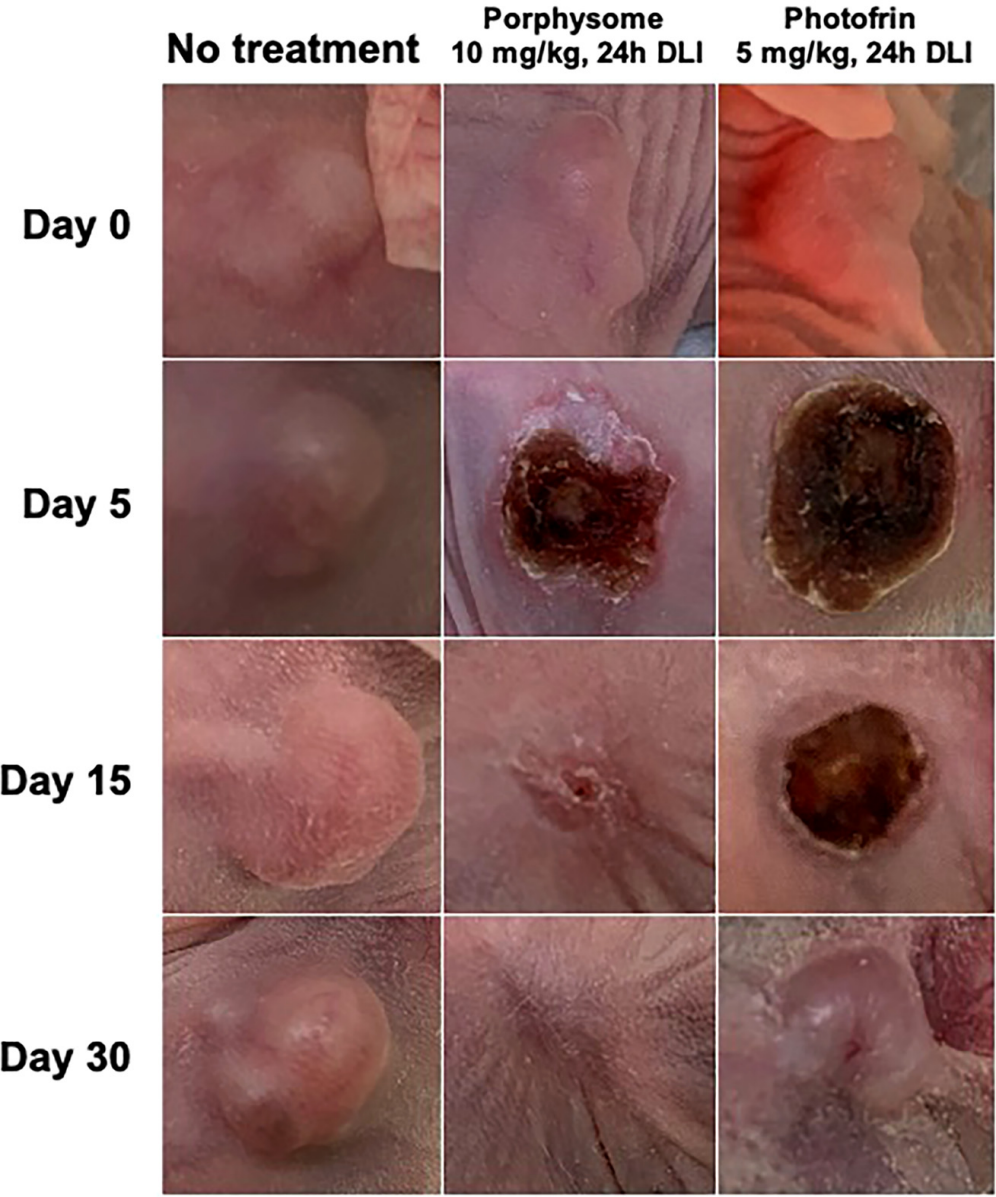
Representative photographs of animal tumors at four time-points (*t* = 0, *t* = 5 days, *t* = 15 days, and *t* = 30 days) in untreated control (left), porphysome-PDT (10 mg/kg, 24 h DLI, middle), and Photofrin-PDT (5 mg/kg, 24 h DLI, right) groups.

Clinically, the area around the tumor became markedly edematous in the first 24–48 h after treatment, which was accompanied by mild lethargy in both the PS-PDT and PHO-PDT groups. These symptoms resolved spontaneously by day 3 post-PDT. Visually, treated tumors responded similarly in both the PS-PDT and PHO-PDT groups, with eschar formation in the first 3–5 days post-treatment, followed by gradual regression of the eschar and healing of the skin between 15- and 30-days following treatment. The visual response to treatment is shown in the representative photograph series seen in Figure 8.

## 4 Discussion

The objectives of this study were to determine whether or not porphysomes are effective PDT photosensitizers, to optimize the PS-PDT treatment regimen, and to compare PS-PDT directly with PHO-PDT. The results show that PSs are comparable in efficacy to the established agent Photofrin. The optimized treatment regimen for PS-PDT, at least in this pre-clinical tumor model, was an *i.v.* dose of 10 mg/kg, 24 h drug-light interval and a light dose of 135 J/cm^2^ (at 100 mW/cm^2^). Using this regimen, a tumor response similar to that of PHO-PDT was achieved, and the rate of complete tumor response approached significance. While the PS dose used, expressed in mg/kg, was twice that of PHO (10 *vs.* 5), the molar doses were comparable, given the molar masses of 1013 [1] and 605.7 g/mol [13], respectively (9.87 × 10^−6^ mol/kg, vs. 8.25 × 10^−6^ mol/kg).

Prior to this study, the effectiveness of unmodified porphysomes for PDT was uncertain. Previous studies had suggested that specific tumor targeting and target-triggered activation (e.g., using folic acid to target folate receptors [8]) were required. The high efficiency of PSs in the intact (optically quenched) nanoparticulate state for photothermal therapy (PTT) was well established in multiple tumor models but it was thought that the concentration of photodynamically-active disrupted pyro-lipids in the tumor may be too low for effective PDT at drug and light doses that would be clinically relevant [1–3, 5–7].

Our previous investigation by Jin et al. [2] investigating the efficacy of PS for PTT did not demonstrate any photodynamic effects; however, this study was not designed to systematically evaluate the PDT potential of PS, and so PDT treatment parameters were not optimized. The most significant difference in treatment between our previous study and the present one is the difference in total light dose (the previous light dose used was 100 J/cm^2^, while the current dose is 135 J/cm^2^). The present study, in combination with previous studies, suggests that PS-PDT is possible at certain light doses, but not others.

Consequently, concurrent PTT and PDT may be possible for the first time using a single all-organic agent and the same light source for both modalities, varying only the energy density between the PDT threshold defined here (100 mW/cm^2^) and the PTT threshold defined in previous studies (1.18 W/cm^2^). Such a regimen is particularly feasible given that the effective PS doses (5–10 mg/kg) and DLI (24 h) are similar to those used in PS-PTT [14]. This dual-modality approach is under active investigation to identify the optimal PS doses and DLIs for each modality and the most effective sequencing of treatments, which is likely to depend on the specific clinical application and tumor type. Thus, for example, one could potentially exploit the immediate “tumor debulking” effect of PTT in combination with the tumor selectivity of PDT at the tumor periphery to give maximum anti-tumoral effect and safety. Combined PDT-PTT may also yield synergistic tumor ablation potential [12]. Previous attempts to create combined PDT-PTT agents have relied on co-delivery of different chromophores (e.g., polydopamine as a photothermal conversion agent, and indocyanine green, IR780, IR820 or other dyes as photodynamic sensitizers), requiring a relatively complex nanoparticle formulation [15], [16]. Such combinations also may or may not require irradiation at different activation wavelengths; the complexity of both the formulation of existing combined PDT-PTT agents and the ultimate delivery of the phototherapy are barriers to translation of these agents that could be overcome with porphysomes [17]. Certain inorganic agents have also demonstrated both PDT and PTT capabilities but have toxicity issues common to inorganic nanoparticles that may limit clinical translation [18], [19]. A critical step in the optimization of combined PS-PDT and PS-PTT is the detailed quantitative determination of the equilibrium dynamics between intact and disrupted PSs in the tumor.

In conclusion, this study has extended the translational potential of the these uniquely multifunctional nanoparticles by adding photodynamic therapy as a potential modality without requiring targeting or other modification of the nanoparticle structure or composition. In addition, it is likely that skin photosensitivity reactions that are a complicating factor in clinical PDT practice may be significantly lower with porphysomes than with Photofrin and this is under active investigation.

## Supplementary Material

Supplementary Material

Supplementary Material
