# Supplementary figures and images for: Porphyrin-lipid nanovesicles (Porphysomes) are effective photosensitizers for photodynamic therapy
Source: Nanophotonics. 2021 Jun 22;10(12):3161–8. doi: 10.1515/nanoph-2021-0220 (PMC9646248; doi:10.1515/nanoph-2021-0220)

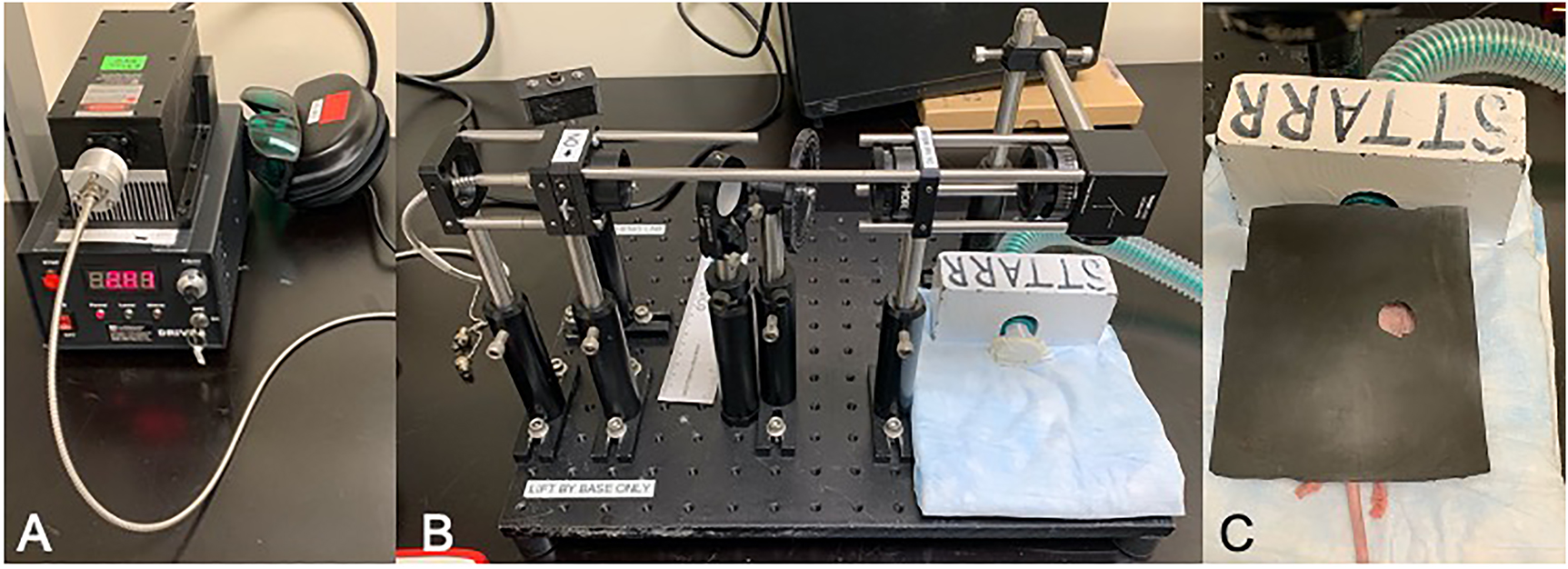

Supplement: Supplementary file 1 — Supplementary Material [file j_nanoph-2021-0220_suppl_001.jpg]

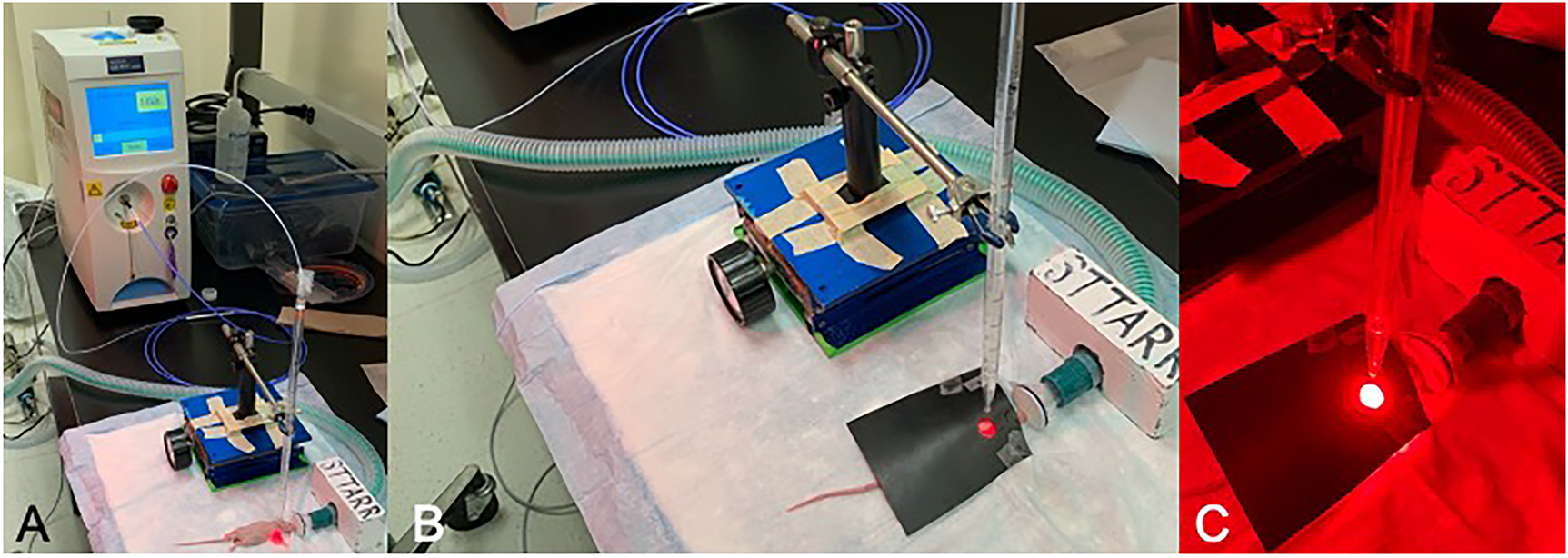

Supplement: Supplementary file 2 — Supplementary Material [file j_nanoph-2021-0220_suppl_002.jpg]
